# Research on Monitoring Exercise-Induced Fatigue Through Infrared Thermal Imaging and Surface Electromyography: A Pilot Study

**DOI:** 10.3390/jfmk11020167

**Published:** 2026-04-23

**Authors:** Hongqiang Liu, Feifei Ma

**Affiliations:** School of Physical Education, Shanxi University, Taiyuan 030006, China; liuhq1969@sxu.edu.cn

**Keywords:** infrared thermal imaging, skin temperature, surface electromyography, exercise-induced fatigue, exercise intensity

## Abstract

**Objectives**: This study aims to investigate the correlations between changes in skin temperature and surface electromyography (sEMG) parameters during fatigue induced by varying exercise intensities. The study uses infrared thermal imaging and sEMG to explore whether skin temperature fluctuations can indicate muscle fatigue states. **Methods**: Two static contraction fatigue tests were administered on the right biceps brachii muscle group of 30 healthy male subjects at 30% and 70% MVC (Maximum Voluntary Contraction) intensity levels. Tests were separated by a 5-day interval and continued until complete fatigue was achieved. The left arm served as a control and was not subjected to any load. Infrared thermal imaging was employed to record continuous skin temperature, capturing data from 120 s pre-exercise to 480 s post-exercise commencement at ten frames per second. Concurrently, sEMG parameters (RMS—Root Mean Square, MPF—Mean Power Frequency, and MF—Median Frequency) were synchronously collected at a sampling frequency of 1 kHz. **Results**: During 70% MVC exercise, skin temperature on the exercised arm consistently decreased, reaching its nadir by the end of the exercise, with a statistically significant divergence from the baseline (*p* < 0.05). At 30% MVC, skin temperature initially slightly declined before gradually increasing. The control arm’s temperature significantly declined across exercise intensities and during recovery. A significant temporal correlation was observed between skin temperature and sEMG parameters. **Conclusions**: 1. Variability in skin temperature patterns during muscular fatigue is contingent on the level of exercise intensity. 2. The strong correlation between skin temperature and sEMG parameters suggests that infrared thermal imaging is a promising, rapid technique for monitoring exercise-induced muscle fatigue.

## 1. Introduction

Muscle fatigue induced by exercise is a physiological phenomenon characterized by a temporary reduction in the muscle’s maximal contraction force or power output [1]. Athletes engage in physical training to facilitate physiological adaptations, ultimately enhancing their athletic performance. An optimal balance between exercise-induced fatigue and recovery is vital for maximizing performance gains [2]. Short-term imbalances can stimulate improvements through a process known as supercompensation [3]; however, persistent fatigue coupled with inadequate recovery can lead to excessive fatigue, thereby compromising athletic performance [4]. Continuous monitoring of an athlete’s fatigue-recovery status can provide valuable feedback, enabling timely adjustments to training regimens and minimizing risks of sports-related injuries and illnesses [5,6].

Surface electromyography (sEMG) has been a primary technique for evaluating muscle fatigue since the 1980s [7,8,9]. However, sEMG has limitations such as electrode displacement and sweat interference, especially during dynamic, high-speed activities. Biochemical assays have also been employed, but these lack specificity in assessing localized muscle fatigue [10,11,12,13]. Traditional physiological measures like lung and heart function tests are unsuitable for evaluating fatigue in specific muscle groups.

Infrared thermal imaging provides a rapid, non-invasive, and wide-field-of-view technique for measuring skin surface temperature by detecting human infrared radiation [14]. Infrared thermography (IRT), as a non-invasive technology, has been widely applied to assess exercise-induced muscle fatigue, diagnose orthopedic conditions, and monitor training loads. In recent years, advancements in device miniaturization, such as smartphone-based thermal imagers, and image analysis techniques have further expanded its potential for application in medical and sports science fields [15,16,17]. During exercise, the body counteracts elevated core temperatures by dissipating heat through dilated skin blood vessels. Therefore, skin temperature changes offer indirect insights into blood flow dynamics during exercise and subsequent recovery [18,19,20].

While some studies have employed infrared thermal imaging to monitor exercise-induced fatigue [21,22,23], most focus solely on temperature changes and athletic performance, neglecting comparisons with other physiological markers like heart rate and sEMG. Surface electromyography (sEMG) and infrared thermography (IRT) are two complementary non-invasive monitoring techniques. sEMG directly reflects the electrophysiological activity of muscles and central drive strategies, while IRT indirectly reveals muscle metabolic heat production, local blood flow perfusion, and autonomic nervous system regulation by detecting changes in skin temperature. Combining these two modalities enables a cross-dimensional assessment of muscle state, from ‘electrical activity’ to ‘thermal effect’. This multimodal approach is not only used in sports science to analyze the mechanisms of muscle fatigue, but also shows potential in the clinical field. Examples include assisting in the evaluation of vasomotor function in complex regional pain syndrome [24], monitoring local inflammatory activity in rheumatoid arthritis [25], and providing objective metrics for post-operative orthopedic rehabilitation assessment [17]. Additionally, limited research has considered temperature variations in limbs on both sides of the body, leading to inconclusive results [26].

Therefore, this study aims to investigate the relationships between skin temperature variations, and exercise intensity (30% MVC, 70% MVC) using an isometric contraction test on the elbow flexor muscles. Infrared thermal imaging will continuously capture temperature data from 120 s pre- and 480 s post-exercise. Simultaneously, sEMG parameters (RMS, MPF, MF) from the exercise arm’s biceps brachii will be collected. This integrative approach seeks to assess the validity of using skin temperature as a reliable marker for muscle fatigue, thereby offering a novel, rapid assessment tool for exercise-induced fatigue monitoring. This study proposed the following hypotheses: (1) The skin temperature of the exercised arm would exhibit distinct dynamic patterns during isometric contraction to exhaustion at different intensities (30% vs. 70% MVC); (2) Changes in the skin temperature of the exercised arm would be significantly correlated with surface electromyography (sEMG) parameters, such as the root mean square (RMS) and median frequency (MF); and (3) Due to neurovascular crossover effects, the skin temperature of the non-exercised control arm would also undergo systematic changes.

## 2. Materials and Methods

### 2.1. Participants

Thirty healthy male university students voluntarily participated in this study. All participants had no regular history of strength training and no history of upper limb musculoskeletal disorders. The mean age, weight, height, and Body Mass Index (BMI) of the participants were 20.6 ± 0.9 years, 65.7 ± 2.8 kg, 180.4 ± 5.8 cm, and 20.2 ± 0.8 kg/m^2^ (M ± SD), respectively. All participants were non-smokers with no history of cardiovascular or pulmonary diseases, and had normal electrocardiograms and arterial blood pressure levels. Additionally, within the last two months, they had not taken medications influencing cardiovascular or thermoregulatory functions. All participants were required to avoid strenuous exercise, prolonged direct sunlight exposure, heavy meals, consumption of alcohol or caffeine, and receiving physical therapy or massage at least 48 h before the test. Additionally, they were instructed to refrain from consuming alcohol or caffeine-containing beverages four hours prior to the experiment. All tests were conducted in the morning to mitigate the impact of circadian rhythms on skin temperature. After comprehending the nature of the experiment, participants gave informed consent. This study was designed as a preliminary pilot investigation. The sample size (*n* = 30) was primarily determined based on feasibility considerations, such as participant recruitment logistics and time/resource constraints. We acknowledge that the lack of an a priori power analysis is a limitation of this study. Future confirmatory studies based on these findings should employ statistical software (e.g., G*Power, version 3.1.9) to perform formal sample size estimation. This study was approved by the Medical Ethics Committee of Shanxi University (Approval Number: SXULL20260092).

### 2.2. Research Design

The study was organized into two distinct phases. In the first phase, the Maximum Voluntary Contraction (MVC) was established, and participants underwent familiarization with isometric contraction tests at intensities of 30% and 70% MVC. The second phase involved fatigue tests at 30% and 70% MVC, separated by a 5-day interval. The right arm served as the test arm, holding a dumbbell with a flexed elbow at 90 degrees until fatigue prevented continuation. The exercise was strictly confined to the sagittal plane, constituting a single-joint (elbow) flexion task, during which participants were required to maintain an elbow joint angle of 90 degrees throughout the contraction period. To control and minimize compensatory movements, the following measures were implemented: (1) The experimenter provided verbal instructions for participants to maintain a neutral wrist position, thereby avoiding wrist flexion or extension; and (2) Throughout the entire testing procedure, an experimenter continuously monitored the participant’s posture. If compensatory movements such as shoulder elevation, trunk lateral flexion, or rotation were observed, immediate verbal cues were given for correction. The left arm acted as a control and remained relaxed.

Before initiating the second phase, participants were required to undertake a standardized warm-up before the second phase, involving a 5-min walk on a treadmill and a 2-min upper limb stretching. Fifteen minutes after acclimatizing to the indoor environment (temperature: 22–23 °C; relative humidity: 50 ± 5%; no direct ventilation or constant bright light), baseline skin temperature and sEMG parameters were collected.

The biceps brachii was selected as the target muscle in this study for the following primary reasons: (1) As the primary elbow flexor, the biceps brachii has an independent function, facilitating standardized isometric testing and straightforward signal interpretation. (2) Its superficial location allows for accurate placement of sEMG electrodes and acquisition of high-quality thermographic images, minimizing excessive interference from deeper tissues or adipose layers. (3) The biceps brachii serves as a canonical model muscle in exercise science for investigating muscle fatigue, force output, and neuromuscular control. Although the present study involved a healthy population, this model establishes a foundation for future application of this methodology to assess individuals with upper limb neuromuscular dysfunctions, such as those post-stroke.

### 2.3. Experimental Equipment

#### 2.3.1. Surface Electromyography (sEMG)

sEMG signals were acquired using the ME6000 P8 system (Mega Electronics, Bittium, Oulu, Finland). Ag/AgCl electrodes were utilized with a contact area of 13.2 mm^2^ and a skin-to-electrode resistance of less than 2 kΩ. The skin was prepared according to Seniam guidelines [27], including hair removal, abrasion, and cleaning with 75% alcohol swabs. Electrodes were placed according to Seniam guidelines, with a sampling frequency of 1000 Hz. Baseline sEMG signals were collected for 120 s before and continuously after exercise until fatigue set in. Parameters like Root Mean Square (RMS) amplitude, Median Frequency (MF), and Mean Power Frequency (MPF) were calculated.

#### 2.3.2. Skin Temperature Measurement

**Figure 1:**
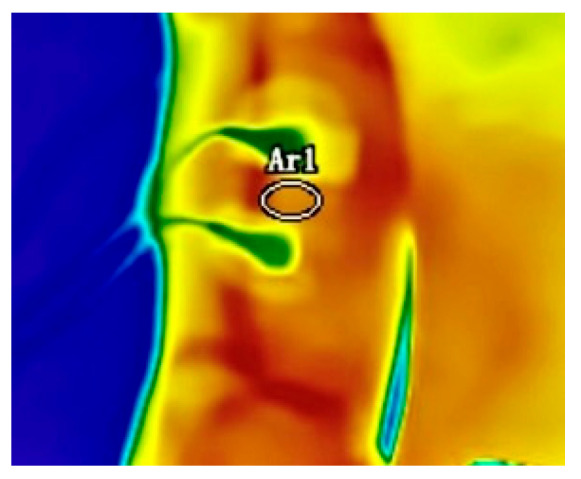
Ellipse Area between Two Electrodes is the Temperature Data Capture Zone.

The skin temperature over the elbow flexor muscles of both arms was recorded using a Fluke Ti480pro infrared thermal camera (Fluke Corporation, Everett, WA, USA). The key technical specifications of the device were as follows: infrared spectral band, 7.5–14 μm; thermal sensitivity (noise equivalent temperature difference, NETD) ≤ 0.05 °C; detector resolution, 640 × 480 pixels (totaling 307,200 data points); imaging frame rate, 60 Hz; calibrated temperature measurement range, −10 °C to 1000 °C with an accuracy of ±2 °C or ±2% of the reading (whichever was greater, at a nominal temperature of 25 °C); spatial resolution (instantaneous field of view, IFOV), 0.93 mRad. The camera was equipped with LaserSharp^®^ (LasX Industries, Inc., White Bear Lake, MN, USA) auto-focus and MultiSharp™ (Fluke Corporation, Everett, WA, USA) multi-focus capabilities to ensure image clarity. During data acquisition, the skin emissivity was set to 0.98 [28]. Participants were positioned at a distance of 1.50 m from the infrared thermographic device against a uniform background. Before exercise initiation, a 120-s thermal imaging sequence was captured to establish baseline skin temperature values. After the commencement of physical activity, a continuous thermal imaging sequence spanning 480 s was captured, incorporating both the exercise and recovery phases. This data was utilized to analyze the fluctuating skin temperature patterns concomitant with muscle contractions. The Fluke Ti480pro device was set to capture 10 thermal frames per second. Thermal image sequences were stored and encoded using Fluke SmartView 4.3 software. To standardize the Region of Interest (ROI), the area between two electrodes on the surface of the biceps brachii was selected, as depicted in Figure 1, following the guidelines stipulated in the Glamorgan Protocol [29]. Subsequently, the mean temperature value for this specific ROI was calculated.

### 2.4. Data Analysis

**Figure 2:**
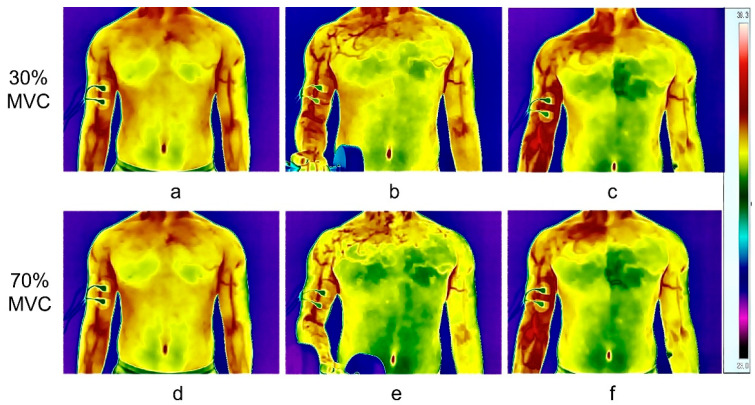
Thermal Images at the Initiation, Termination and Recovery Phases across (**a**–**c**) 30% and (**d**–**f**) 70% MVC Intensity.

**Figure 3:**
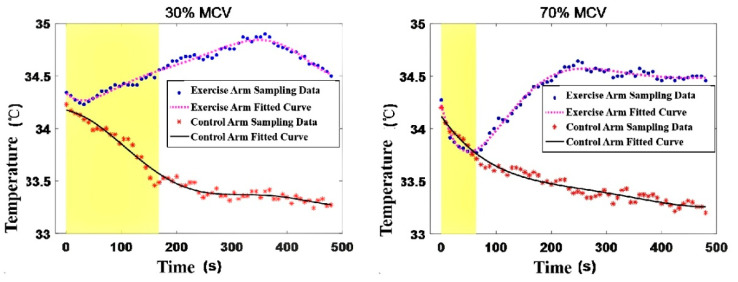
Trends in Skin Temperature Variations During Tests at 30% MVC and 70% MVC.

**Figure 4:**
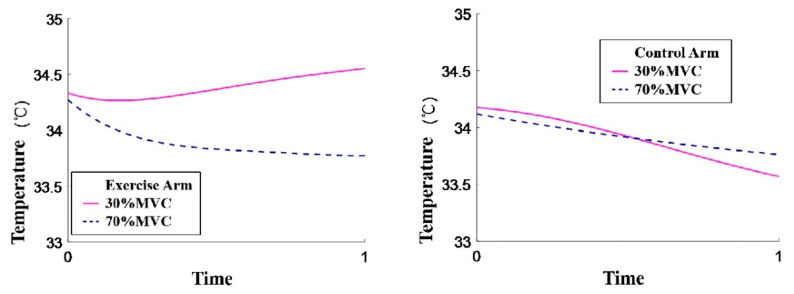
Trends in Skin Temperature Changes During the Exercise Phase.

This research aims to investigate (a) the influence of exercise intensity on skin temperature variations and (b) the correlation between skin temperature and electromyographic parameters (RMS, MF, and MPF) during exercise. Temperature data were collected at different phases: initiation, termination, and recovery, as illustrated in Figure 2. Baseline skin temperatures were determined by calculating the average temperature over 120 s before exercise commencement. Minimum and maximum temperatures are denoted as “nadir” and “zenith” respectively. Matlab (R2026a) was employed for Fourier series fitting to analyze the pattern of skin temperature fluctuations in both the exercise and control arms during the three aforementioned phases. Figure 3 showcases the resulting fit curve, while Figure 4 illustrates the normalized time trends for skin temperature variations in both arms.

Key sEMG parameters, including RMS, MF, and MPF, were calculated and subsequently correlated with skin temperature (T). Baseline values for these electromyographic measures were established by averaging data from the initial 120 s prior to exercise onset.

The statistical examination presents data as medians and first and third quartiles, i.e., Median (Q1, Q3). The normality of parameter distribution is assessed using graphical methods (histograms and normal probability plots) and the Shapiro–Wilk test. Aside from the baseline temperature, none of the other parameters adhered to the normal distribution assumption. Repeated-measures ANOVA is employed to analyze baseline temperature, whereas Wilcoxon signed-rank tests are utilized for the remaining parameters. The relationship between skin temperature and electromyographic metrics is assessed using the Spearman rank correlation coefficient. Statistical analyses are conducted using SPSS 26.0 software, with the significant level α = 0.05. Comparative analyses of baseline temperature, end-of-exercise temperature, end-of-recovery temperature, and nadir values during the same exercise and recovery phases are shown in Table 1. Comparative analyses of various parameters under different intensities during the exercise and recovery phases are displayed in Table 2.

## 3. Results

Table 1 compares skin temperatures between the two arms under identical exercise conditions. In conjunction with Figure 3, it is observed that the nadir skin temperature in the exercise arm occurs early in the exercise at 30% MVC and at the termination of exercise at 70% MVC. Table 2 offers a comparative analysis of parameters between the exercise and control arms across different intensities.

**Figure 5:**
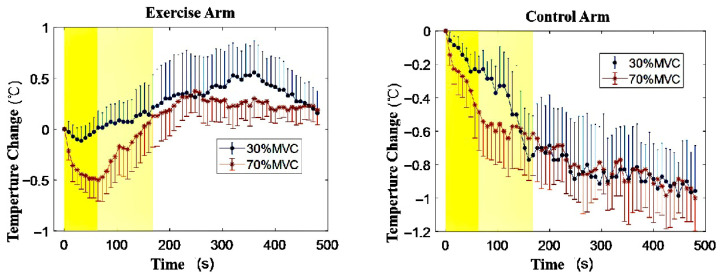
Mean Temperature Changes During Exercise and Recovery for 30% MVC and 70% MVC.

Overall, skin temperature variations in the exercise arm under two different intensities during exercise and recovery demonstrate the following characteristics:

- During the exercise-to-fatigue process at 30% MVC, the skin temperature of the biceps brachii displays an initial minor decrease followed by a gradual rise. At 70% MVC, skin temperature reveals a progressively declining trend and a significant difference is observed when compared to baseline temperature upon exercise termination (*p* < 0.05).
- In the initial 30 s of exercise commencement, a skin temperature drop is evident under both exercise intensities, with a significant variation in temperature change (*p* = 0.042). The decline in skin temperature is more substantial and occurs at a faster rate at 70% MVC (as depicted in Figure 5).
- Compared to the 30% MVC, muscle fatigue occurs more readily, and endurance time is significantly reduced at 70% MVC (*p* = 0.018).
- During the recovery phase, skin temperature in both exercise intensities rises (exceeding baseline levels) and subsequently decreases, converging towards the baseline temperature. The skin temperature in the control arm exhibits a similar trend in both intensities, progressively declining throughout the entire exercise and recovery process.

The relationship between skin temperature (T) and sEMG parameters (RMS, MPF, and MF) during exercise was assessed through Spearman correlation. Table 3 presents the correlation coefficients and significance levels for parameters T, RMS, MPF, and MF. The analysis indicates a statistically significant relationship between biceps brachii skin temperature and sEMG parameters during exercise at both 30% MVC and 70% MVC.

## 4. Discussion

This study aimed to investigate the dynamic changes in skin temperature of the anterior arm and their association with neuromuscular activity during isometric elbow flexion to exhaustion at different intensities in healthy adults, utilizing infrared thermography (IRT) and surface electromyography (sEMG). The principal findings were as follows: (1) The pattern of skin temperature change in the exercised arm was intensity-dependent, exhibiting an initial slight decrease followed by a gradual increase at 30% MVC, whereas a progressive decline to a level significantly below baseline was observed at 70% MVC. (2) Significant correlations were found between the skin temperature of the exercised arm and sEMG parameters (RMS and MF), although the direction of these correlations differed with exercise intensity. (3) The skin temperature of the non-exercised control arm demonstrated a progressive decrease during the exercise period, suggesting the potential presence of a crossover effect. The following sections will discuss these findings in depth.

### 4.1. Analysis of Skin Temperature Variation in the Exercise Arm

Initially, in tests conducted at 30% MVC and 70% MVC, the subjects reached a state of complete fatigue, yet significant differences were observed in the change in skin temperature of the exercise arm. These differences can be attributed to the impact of varying exercise intensities and durations on peripheral vasoconstriction. Monitoring the skin temperature changes during the initial 30 s of activity is crucial, as these changes are independent of the duration of the two activities and display the most notable differences within this period. Although the data does not delineate the individual effects of exercise intensity and duration on skin temperature changes, it does suggest that 30% MVC and 70% MVC exercise intensities have disparate overall impacts on the thermoregulatory systems controlling skin blood flow and heat dissipation.

Skin temperature variation indirectly reflects alterations in muscular blood flow levels. Blood circulation serves as the primary medium for transferring core body heat to the skin layer. Under this mechanism, skin blood flow can undergo significant changes to dissipate heat or conserve it, resulting in noticeable temperature fluctuations [30,31]. Current literature supports the findings of this study regarding the skin temperature variations in the load-bearing muscles during 30% MVC and 70% MVC activities [32]. However, these previous studies did not utilize sEMG or heart rate as physiological indicators for correlation. Future research could consider combining infrared thermography and blood flow measurement devices to validate muscular blood flow during 30% MVC and 70% MVC activities.

Furthermore, the rate and extent of skin temperature decrease in the biceps brachii during 70% MVC exercise were noticeably higher than during 30% MVC, as depicted in Figure 5. This phenomenon reflects differing skin blood flow responses under different intensities. Rowell et al. [33] have attributed the ongoing decline in skin blood flow during exercise to a consistent increase in vasoconstricting catecholamines and other hormones. The difference in the rate and degree of temperature drop could be related to exercise intensity. Other research suggests that muscle blood flow increases in proportion to the activation and recruitment of motor units, and the rise in muscle temperature enhances enzyme activities, allowing for immediate blood flow into stressed muscles during intense exercise [34]. Alvares et al. [35] examined the effects of various speeds of isokinetic exercise on femoral artery blood flow, hemoglobin, and oxygen saturation levels. The findings can be used to explain why skin temperature decreases more noticeably at the onset of high-intensity exercise than low-intensity exercise. Unlike low-intensity, long-duration exercise, muscle fatigue during high-intensity, short-duration exercise is challenging to alleviate during the activity and only quickly recovers once the load is removed.

### 4.2. Analysis of Skin Temperature Variation in the Control Arm

Throughout physical exertion and subsequent recovery, the skin temperature of the control arm demonstrated a consistent trend of gradual decline. Statistically significant differences were observed when comparing the skin temperature at the end of exertion and during recovery to baseline levels. Exercise has been shown to induce systemic endothelial adaptations, influencing the vasculature of the muscles directly engaged in activity and that of muscles not directly participating [36,37]. This phenomenon may account for the observed reduction in skin temperature in the control arm. Moreover, studies by Bandeira et al. [38] have indicated that the sympathetic nervous system modulates skeletal muscle vasculature. During physical activity, the sympathetic system directs blood flow from the control limb to the active limb, providing another plausible explanation for the decline in skin temperature of the control arm. Moreover, it has been suggested that unilateral limb exercise or fatigue can systemically influence the vasomotor tone or metabolic state of the contralateral limb via central nervous system or neuroendocrine pathways [16]. For instance, Cabizosu et al. found that national-level sprinters exhibited significant thermoregulatory changes not only in the exercised limb but also in the myotendinous region of the contralateral limb following a unilateral fatiguing bout [16]. This supports the concept that unilateral exercise stress can elicit bilateral physiological responses. Therefore, the temperature decrease observed in the control arm in the present study likely reflects a systemic sympathetic excitation or blood flow redistribution triggered by fatigue in the exercised side, rather than being caused by local factors. Previous research investigating unilateral arm contraction has primarily ignored the analysis of the control arm, except for one study by Neves et al. [24], which produced results consistent with the present study. Exercise induces a redistribution of blood flow in the body, particularly to meet the metabolic demands of active skeletal muscles [39,40], thus affecting skin temperature. This trend is graphically represented in Figure 2, which illustrates the gradation in skin temperature from the control side to the active side.

### 4.3. Correlation Analysis Between Skin Temperature Changes and sEMG Parameters

This investigation probed the relationship between neuro-muscular activation of the biceps brachii during isometric contraction fatigue tests and corresponding skin temperature variations. Data were collected via sEMG and were correlated with skin temperature changes recorded synchronously through infrared thermography. Statistical analyses revealed a significant correlation between all evaluated sEMG parameters (RMS, MPF, and MF) and variations in skin temperature over time. The progressive increase in temperature variations may be attributable to muscle fatigue. Given the notable correlation between skin temperature and sEMG parameters, which serve as critical indices for muscle fatigue detection, it is inferred that bicep brachii fatigue significantly influenced the observed cutaneous temperature changes.

The captured sEMG parameters are susceptible to subcutaneous influences, and similarly, thermal readings in infrared thermography are not exempt from subcutaneous modulations. Specifically, the recorded temperatures are mediated by adipose tissue between the skin and underlying musculature. Ruiter et al. [41] employed thermocouples to record surface skin temperatures and estimated intramuscular temperatures based on a linear relationship between skin and muscle temperatures. Such indirect monitoring of muscular temperature can, to some extent, mitigate the onset of muscle injury and prevent excessive fatigue [16].

### 4.4. Advantages of Infrared Thermographic Imaging in Fatigue Monitoring

The benefits of infrared thermographic imaging are manifest in several domains, including rapid data acquisition, intuitive visualization, extensive field of view, non-contact testing, and ease of data capture. Infrared thermography offers greater convenience and applicability than existing modalities for assessing muscle fatigue, such as electromyographic (EMG) techniques, blood biochemistry, cardiac function, and pulmonary function tests. The technology allows real-time monitoring of localized or systemic fatigue states through skin temperature fluctuations, offering invaluable insights into localized fatigue—a latent cause of bodily injury [42,43,44]. Substantial alterations in localized skin temperature are readily discernible through thermal imaging. As illustrated in Figure 2, the temperature of the right biceps brachii persists in elevating post-exertion, failing to revert to baseline even after eight minutes, while the temperature of the contralateral muscle maintains a lower value. Thus, monitoring surface skin temperature could serve as a pragmatic index for training optimization and injury prevention. Additional research has indicated that post-exercise body surface cooling mitigates thermal discomfort, accelerates the reversion of core body temperature to pre-exercise levels, and facilitates energy conservation, promoting rapid recovery [45].

Another noteworthy finding of this study was the high degree of symmetry (difference < 0.5 °C) observed in the skin temperature of both arms in healthy participants under strictly controlled baseline conditions. This outcome not only validates the effectiveness of our participant screening, environmental acclimatization, and pre-test standardization protocols, but also underscores the importance of establishing stringent control conditions in thermographic research. This baseline symmetry provides a physiological rationale for utilizing the non-exercised limb as a reliable internal control. Future research can build upon this foundation to explore whether bilateral skin temperature asymmetry could serve as a sensitive and objective assessment metric in patient populations, such as those with unilateral injuries, post-stroke hemiparesis, or complex regional pain syndrome.

The preliminary findings of this study possess multifaceted potential applications: (1) Sports training monitoring: The combination of IRT and sEMG can provide coaches with more comprehensive feedback on athlete status for optimizing training intensity and preventing overtraining. (2) Rehabilitation medicine assessment: It offers an objective and non-invasive tool for evaluating neuromuscular function and fatigue in patients with upper limb dysfunction caused by conditions such as stroke or spinal cord injury, useful for monitoring rehabilitation progress. (3) Clinical auxiliary diagnosis: It serves as an adjunctive means for assessing local vasomotor function and inflammatory status in diseases like Complex Regional Pain Syndrome and rheumatoid arthritis. (4) Ergonomics: It can be used to quantify muscle load during static or repetitive tasks, providing a basis for workstation design and work-rest schedule arrangements.

### 4.5. Limitations and Future Directions

Despite selecting participants with closely matched Body Mass Index (BMI) to minimize the impact of subcutaneous fat on the study [46], its influence on skin temperature and sEMG parameters could not be entirely negated. The sample size was insufficient; future studies should incorporate a larger cohort. Although environmental factors affecting skin temperature were considered, the study was not conducted in a controlled environment, suggesting higher standards for future research. The current study was confined to investigating temperature changes and electromyographic signals during isometric contractions until fatigue in localized areas. Future investigations should expand the temperature sampling zones and examine different contraction modalities. This study exclusively recruited a cohort of healthy young males, thereby not accounting for the potential influences of sex, age, or basal metabolic differences on the outcomes. Future research is required to include broader or more specific populations to test the generalizability of the current findings and to explore the moderating role of these demographic factors on exercise-induced thermal responses and fatigue characteristics. It is important to note that the infrared thermal camera used in this study was a research-grade device. It was not specifically designed for clinical medical diagnosis, nor is it approved by regulatory bodies such as the U.S. Food and Drug Administration (FDA) for the diagnosis of human diseases Consequently, the findings should be interpreted as an exploratory description of physiological phenomena. Future applications of infrared thermography for clinical assessment or diagnosis would necessitate the use of medically certified equipment and the establishment of corresponding clinical diagnostic standards.

## 5. Conclusions

The findings of this preliminary study indicate that during isometric contractions to exhaustion at different intensities, the skin temperature of the arm exhibits an intensity-dependent dynamic pattern and shows correlation with sEMG parameters, and a possible cross-over effect was observed on the non-exercising side. These exploratory findings suggest that combining infrared thermography with surface electromyography may offer a multi-dimensional perspective for assessing muscle fatigue and neurovascular responses. However, given the limited sample size and pilot nature of this study, these conclusions require validation through larger-scale and more rigorously designed research. Future work should focus on standardizing testing protocols, incorporating more diverse populations, and exploring its application efficacy in specific clinical and athletic contexts.

## Figures and Tables

**Table 1:** Comparative Analysis of Parameters During Exercise and Recovery Phases.

Parameter (°C)	30% MVC			70% MVC		
	Exercise Arm	Control Arm	*p*	Exercise Arm	Control Arm	*p*
Baseline Temp	34.4 (33.9, 34.7)	34 (33.8, 34.9)	0.334	34.3 (34.1, 34.5)	34.2 (34.1, 34.4)	0.14
End Temp	34.7 (34.5, 35.3)	33.5 (33.3, 33.6) *	0.018	33.5 (33.5, 34) *	33.8 (33.4, 34) *	0.581
Recovery End Temp	34.7 (34.4, 34.7)	33.2 (33.1, 33.5) *	0.016	34.4 (34.2, 34.7)	33.4 (33.2, 33.7) *	0.018
Nadir Value	34.2 (33.8, 34.4)	——	——	33.5 (33.5, 34) *	——	——

Note: Values are presented as medians and first and third quartiles, i.e., Median (Q1, Q3). Repeated-measures ANOVA is used for baseline temperature, whereas the Wilcoxon signed-rank test is employed for other parameters. * indicates statistical significance compared to baseline temperature.

**Table 2:** Comparative Analysis of Various Parameters Across Exercise and Recovery Phases.

Parameters	30% MVC	70% MVC	*p*
Exercise Duration (s)	168 (144, 184)	58 (56, 80)	0.018
Baseline Temp of Exercise Arm (°C)	34.4 (33.9, 34.7)	34.3 (34.1, 34.5)	0.754
End Temp of Exercise Arm (°C)	34.7 (34.5, 35.3)	33.5 (33.5, 34)	0.017
Temp Change at Exercise End (Exercise Arm, °C)	0.4 (0.2, 0.7)	−0.6 (−1.1, −0.3)	0.018
Temp at Recovery End (Exercise Arm, °C)	34.7 (34.4, 34.7)	34.4 (34.2, 34.7)	0.752
Baseline Temp of Control Arm (°C)	34 (33.8, 34.9)	34.2 (34.1, 34.4)	0.897
End Temp of Control Arm (°C)	33.5 (33.3, 33.6)	33.8 (33.4, 34)	0.400
Temp at Recovery End (Control Arm, °C)	33.2 (33.1, 33.5)	33.4 (33.2, 33.7)	0.610
Temp Change at 30 s (Exercise Arm, °C)	−0.1 (−0.2, −0.1)	−0.4 (−0.8, −0.2)	0.042
Peak Temp (°C)	35.1 (34.9, 35.3) *	34.6 (34.4, 34.8)	0.310

Note: Values represent the median and first and third quartiles (Median [Q1, Q3]). Repeated-measures ANOVA is employed for baseline temperature comparisons, while the Wilcoxon signed-rank test is used for the analysis of other parameters. * indicates statistically significant differences compared to the baseline temperature.

**Table 3:** Spearman Correlation Analysis of Skin Temperature (T) and sEMG Parameters (RMS, MPF, and MF) During Exercise.

	RMS		MPF		MF	
	R	*p*	R	*p*	R	*p*
30% MVC	0.32	0.012	−0.333	0.009	−0.284	0.027
70% MVC	−0.272	0.035	0.41	0.001	0.335	0.008

## Data Availability

The datasets used and/or analyzed during the current study are available from the corresponding author on reasonable request.
